# Clinical Lectures on Diseases of the Eye and Ear, Etc.

**Published:** 1868-09-15

**Authors:** 


### CLINICAL LECTURES ON DISEASES OF THE EYE AND EAR, AT THE CHICAGO CHARITABLE EYE AND EAR INFIRMARY.

The Sixth Course of Clinical Lectures at the Infirmary (16 East Pearson street), by Dr. E. L. Holmes, will commence October 5th, at half-past one o'clock, P.M., and continue twenty weeks, on such days and at such hours as may be found most convenient to those who attend.

No Institution in the Northwest offers the student and practitioner superior opportunities for the clinical study of all forms of ophthalmic diseases and their medical and surgical treatment.

During the past year, more than 800 patients have received the benefits of the institution, of whom a large number required important surgical operations on the eye.

During the last course, there was an average daily attendance of 40 patients at the Infirmary.

Excellent opportunities will be afforded of studying each case, and comparing it with other cases. The abnormal condition of the various tissues of the eye will be illustrated, not only by the cases under treatment, but also by numerous plates, and by a large number of pathological specimens. Instruction will also be given in the use of the ophthalmoscope, and in the best modes of examining the ear.

Certificates of attendance will be given to the members of the class, at the close of the course.

Tickets for the course will be $5.00 each. The fees will be devoted to the support of the Infirmary.

